# Proteinuria among Pregnant Women Admitted to the Department of Obstetrics and Gynaecology of a Tertiary Care Centre

**DOI:** 10.31729/jnma.8388

**Published:** 2024-01-31

**Authors:** Asmita Ghimire, Poonam Koirala, Hima Rijal, Anita Chamlagain, Padam Raj Pant

**Affiliations:** 1 Department of Obstetrics and Gynaecology, Tribhuvan University Teaching Hospital, Maharajgunj, Kathmandu, Nepal

**Keywords:** *preeclampsia*, *pregnancy complications*, *proteinuria*

## Abstract

**Introduction:**

Proteinuria is usually related with preeclampsia during pregnancy, although it can also be caused by other conditions such as renal disease or isolated proteinuria. Proteinuria during pregnancy can result in adverse fetomatemal outcomes. The study aimed to find the prevalence of proteinuria among pregnant women admitted to the Department of Obstetrics and Gynaecology of a tertiary care centre.

**Methods:**

A descriptive cross-sectional study was carried out among pregnant women in the Department of Obstetrics and Gynaecology after obtaining ethical approval from the Institutional Review Committee. Data of 14 April 2022 to 13 April 2023 was collected from 9 June 2023 to 9 September 2023 from medical records. The study included pregnant women aged 18-45 years, who were past 28 weeks of gestation and had a 24-hour urine protein measurement. Pregnant women who had insufficient medical records were excluded from the study. A convenience sampling method was used. The point estimate was calculated at a 95% Confidence Interval.

**Results:**

Among 3,914 pregnant women, proteinuria was seen in 61 (1.56%) (1.17-1.95, 95% Confidence Interval). The mean proteinuria in the study group was 1.5±2.75 gm/24 hr. In pregnant women with proteinuria, maternal complications were seen in 51 (83.60%) and foetal complications in 34 (55.73%) cases. A total of 47 (77.05%) underwent emergency lower-section caesarean section.

**Conclusions:**

The prevalence of proteinuria among pregnant women was found to be similar as compared to studies done in similar settings.

## INTRODUCTION

Proteinuria in pregnancy is defined as the appearance of protein in the urine in amounts equal to or greater than 300 mg of protein in 24-hour collection, protein/creatinine ratio equal to or greater than 0.3 mg, or +2 or more on urine dipstick testing.^1,2^ Preeclampsia is an important cause of proteinuria during pregnancy with its incidence ranging from 2-8%. Nephrotic range proteinuria which is more severe than nephritic range proteinuria is reported to occur in 0.012-0.025% of all pregnancies.^3^ The gold standard for quantification of proteinuria is the 24-hour urine protein collection.^4^

Proteinuria in pregnancy can lead to adverse fetomaternal outcomes. It may lead to severe hypertension, raised liver enzymes, renal insufficiency, thrombocytopenia, admission to the intensive care unit and mortality.^5^

The study aimed to find the prevalence of proteinuria among pregnant women admitted to the Department of Obstetrics and Gynaecology of a tertiary care centre.

## METHODS

A descriptive cross-sectional study was conducted among pregnant women admitted to the Department of Obstetrics and Gynaecology in Tribhuvan University Teaching Hospital (TUTH), Maharajgunj, Kathmandu, Nepal after obtaining ethical approval from the Institutional Review Committee of the same institute (Reference number: 562(6-11)E^2^). Data of 14 April 2022 to 13 April 2023 was collected from 9 June 2023 to 9 September 2023 from medical records. The study included all pregnant women with >28 weeks gestation period in the age group 18-45 years who had undergone 24 hours of urinary protein assessment. Pregnant women who had insufficient medical records regarding prenatal records, laboratory results, obstetrics history, delivery outcomes, neonatal outcomes, and severe comorbidities that affect fetomaternal outcomes like severe cardiovascular disease, and autoimmune disease were excluded from the study. A convenience sampling method was used. The sample size was calculated using the following formula:


$$\begin{array}{ll} \text{n} & ={\text{Z}}^{2}\times \frac{\text{p}\times \text{q}}{{\text{e}}^{\text{2}}}\\ & =1.96^{2}\times \frac{0.50\times 0.50}{0.02^{2}}\\ & =2,401 \end{array}$$

Where,

n = minimum required sample size
Z = 1.96 at 95 % Confidence Interval (CI)
p = prevalence taken as 50% for maximum sample size calculation
q = 1-p
e = margin of error, 2%

The calculated sample size was 2,401. However, a total of 3,914 samples were taken.

All women who had been admitted and delivered in TUTH during the study period were recorded using the record book of the labour room, maternity ward, female surgical ward and medical record section. Information about maternal risk factors, maternal complications and obstetric outcomes were noted in performa. Information about neonatal complications was obtained from the record book of the labour room, neonatal unit and neonatal intensive care unit (NICU). The 24 hour urine protein analysis was the standard test for diagnosis of proteinuria. Degrees of proteinuria can be classified as normal (150 mg/24 hr), nephritic (150-3000 mg/24 hr) and nephrotic (3500 mg/24 hour).^6^ So taking this as standard, >150 mg/dL was considered as abnormal proteinuria during pregnancy.

Data was entered into Epi Info and analysis was done using IBM SPSS Statistics version 22.0. The point estimate was calculated at a 95% CI.

## RESULTS

Among 3,914 pregnant women, proteinuria was seen in 61 (1.56%) (1.36-1.76, 95% CI). The mean proteinuria in the study group was 1.5±2.75 gm/24 hr. The mean age of the pregnant women with proteinuria was 30.13±4.39 years (Table 1).

**Table 1:** General characteristics of pregnant women with proteinuria (n= 61).

Parameters	Mean±SD
Age (years)	30.13±4.39
Period of gestation (weeks)	35.96±2.50
Body mass index (kg/m^2^)	26.3±4.49

More than one-third of the patients, 24 (39.34%) had mild preeclampsia (Table 2).

**Table 2:** Causes of proteinuria (n= 61).

Causes	n (%)
Mild preeclampsia	24 (39.34)
Severe preeclampsia	13 (21.31)
Chronic hypertension	13 (21.31)
Superimposed preeclampsia	4 (6.56)
Impending eclampsia	4 (6.56)
Eclampsia	1 (1.64)
IgA nephropathy	1 (1.64)
SLE nephritis	1 (1.64)

Maternal complications were seen in 51 (83.60%) of the patients among whom all had raised liver enzymes. A total of 34 (55.73%) newborns were found to have intra uterine growth retardation (IUGR) (Table 3).

**Table 3:** Fetomaternal complications (n = 61).

Parameters	n (%)
Maternal complications	51 (83.60)
Raised liver enzymes	51 (83.60)
Thrombocytopenia	16 (26.23)
Raised renal enzymes	2 (3.28)
Neurological toxicity	1 (1.64)
**Fetal complications**	**34 (55.73)**
Intrauterine growth restriction	34 (55.73)
Respiratory distress syndrome	6 (9.84)
NICU	6 (9.84)
Very preterm	2 (3.28)
Early moderate preterm	9 (14.74)
Late moderate preterm	20 (32.78)

Among the pregnant women with preeclampsia, 52 (85.25%) underwent lower section caesarean section (LSCS) of which 47 (77.05%) was emergency LSCS (Table 4).

**Table 4:** Mode of delivery (n= 61).

Parameters	n (%)
Vaginal delivery	9 (14.75)
Spontaneous	1 (1.64)
Induced	7 (11.47)
Induced Operative Vaginal delivery	1 (1.64)
**LSCS**	**52 (85.25)**
Emergency	47 (77.05)
Elective	5 (8.20)

Foetal distress was an indication of LSCS in 17 (32.69%) patients (Table 5).

**Table 5:** Indications for LSCS (n = 52).

Indications	n (%)
Foetal distress	17 (32.69)
Cephalopelvic disproportion	2 (3.85)
Non-progress of labour	5 (9.62)
Previous LSCS	5 (9.62)
Breech	3 (5.77)
Uncontrolled hypertension	9 (17.31)
Oligohydramnios	5 (9.62)
Abnormal doppler	4 (7.69)
Bad obstetric history with antiphospholipid syndrome	1 (1.92)
Scar tenderness with previous LSCS	1 (1.92)

## DISCUSSION

In this study, the prevalence of proteinuria among pregnant women was found to be 1.56%. In a similar study, the prevalence of proteinuria was found to be 3.8%.^7^ Proteinuria during pregnancy has been the topic of multiple research, all of which have consistently linked it to poor maternal and foetal outcomes. In a foetus, it can lead to intrauterine foetal death, stillbirth, preterm delivery, intrauterine growth restriction, neonatal intensive care unit stay and neonatal death.^2,3,5,8^ The relationship between proteinuria and fetomaternal outcomes has been the subject of research.

The protein/creatinine ratio in random urine samples was greater in primigravida women (0.66) than in multigravida women (0.36), according to a study done in 150 pregnant women with preeclampsia in Iran.^9^ This study, however, did not employ 24-hour urine protein samples. The present study where a 24-hour urinary sample was done to assess proteinuria showed more number of primigravidae patients. Proteinuria is a common symptom of pregnancy, and one of the most prevalent causes of proteinuria in pregnancy is preeclampsia similar to the present study.^10^

Proteinuria in pregnancy might vary in intensity and specific complications depending on individual circumstances and underlying health conditions, according to a retrospective study done in 239 pregnant preeclamptic women in China.^11^ Similarly, the current study also found that increased maternal proteinuria problems, such as elevated liver enzymes, elevated renal enzymes, and thrombocytopenia, were more common. This emphasises the significance of routine antenatal screening and monitoring in detecting and managing possible proteinuria issues.

Another retrospective study done in 165 women with isolated gestational proteinuria published in Acta Obstet Gynecol Scand in the year 2021 found that proteinuria during pregnancy is associated with an increased risk of preterm birth and small-for- gestational-age newborns.^12^ In comparison, this study also found that mothers with proteinuria had a greater rate of preterm deliveries and SGA infants. This implies that the intensity of proteinuria is connected to the severity of preeclampsia and its sequelae.

Several studies, including those done in 162 pregnant Chinese population, have found that proteinuria during pregnancy is linked to adverse pregnancy outcomes such as preterm labour, premature rupture of membranes (PROM), IUGR, and preeclampsia.^13^ Foetal problems like IUGR, respiratory distress syndrome, NICU stay, and prematurity were also reported in the present study. While the specific issues and degree of proteinuria might vary depending on individual circumstances and underlying health disorders, it is critical to emphasise the significance of frequent antenatal screening and treatment and monitoring to detect and manage any potential proteinuria problems.

A review article published in 2012, recommended that the decision to perform a caesarean section in women with preeclampsia should be based on the severity of the condition and the presence of other risk factors.^14^ The current study, which found a higher prevalence of fetomaternal problems, also found a higher percentage of caesarean section deliveries in 52 (82.25%). This means that the decision to perform a caesarean section should be taken on a case-by-case basis, taking into account the specific circumstances of each patient.

A published cohort study among 11651 women in the UK in the year 2011 established that higher maternal pre-pregnancy BMI was associated with an increased risk of proteinuria during pregnancy.^15^ The mean BMI of pregnant women with proteinuria in our study was 26.3±4.49 kg/m^2^.

Limitations of this study include reliance on medical record data and limited generalizability. Additionally, it is recommended to conduct an analytical study with a focus on the association between proteinuria and adverse maternal and fetal outcomes.

## CONCLUSIONS

The prevalence of proteinuria among pregnant women was found to be similar to studies done in similar settings. Antenatal screening, monitoring and management of proteinuria could be done in high risk pregnant women to prevent complications associated with proteinuria.
